# Training needs assessment of veterinary practitioners in Ethiopia

**DOI:** 10.1007/s11250-022-03075-0

**Published:** 2022-01-22

**Authors:** Ruth Alafiatayo, Erika Galipo, Abel B. Ekiri, Mariana Dineva, Isabella Endacott, Tewodros Tesfaye, Gewado Gellebo, Fasil Awol, Erik Mijten, Gabriel Varga, Alasdair J. C. Cook

**Affiliations:** 1 Department of Veterinary Epidemiology and Public Health, School of Veterinary Medicine, Faculty of Health and Medical Sciences, University of Surrey, Guildford, UK; 2 Ethiopia Veterinary Association, Addis Ababa, Ethiopia; 3 Zoetis-ALPHA Initiative, Zoetis, Zaventem, Belgium

**Keywords:** Ethiopia, Livestock, Training, Animal health, Veterinarian, Veterinary professionals, CPD

## Abstract

Pastoral and agro-pastoral farming are extensively practised in Ethiopia, and the main livestock kept are cattle, goats, sheep, poultry, and camels. The livestock sector is faced with complex challenges including limited availability of well-trained and skilled animal health professionals. The objective of this study was to identify and prioritise areas for training with the goal of providing evidence to guide strategies to improve the skills, delivery, and governance of veterinary services across Ethiopia. A cross-sectional survey was developed and administered electronically to veterinary professionals in Ethiopia using the Qualtrics platform. Data were collected on select parameters including demographics, diseases of economic significance, diagnosis, disease prevention, biosecurity, disease control, treatment, epidemiology, One Health, disease reporting, and the participants’ opinions about training. The survey data was downloaded in Microsoft Excel and descriptive statistics performed. A total of 234 veterinary professionals completed the survey. Most participants were male (89.7%) and aged between 26 and 35 years (81.2%). Of the total respondents, 56.4% worked in government and 8.5% in private practice. Most participants perceived training on laboratory diagnostic testing, disease prevention, antimicrobial resistance, antibiotic sensitivity testing, basic epidemiology, and clinical procedures, as most beneficial. In addition, most respondents would like to receive training on diseases affecting cattle, poultry, and small ruminants. The findings from this study provide baseline information on priority training areas for veterinary professionals and could potentially contribute to national efforts to develop and implement a continuing professional development programme in the veterinary domain, in view of improving veterinary service delivery.

**Supplementary Information:**

The online version contains supplementary material available at 10.1007/s11250-022-03075-0.

## Introduction

Pastoral and agro-pastoral farming are extensively practised in over 60% of the land area in Ethiopia. The main livelihood of these farmers is from the production of livestock, mainly cattle, goats, sheep, poultry, and camels. Livestock are critical to the wellbeing of households in terms of income, savings, food security, and employment. The livestock sector is also important to the national economy, contributing 16% of the total GDP, 33% of agricultural GDP, and 8% of export earnings. Improvements in farming have the potential to contribute significantly to national income and the welfare of many poor families (Admassu, 2010; CSA 2016).

The national herd in Ethiopia consists of about 55.2 million cattle, 29 million sheep and the same number of goats, 4.5 million camels, and about 50 million poultry (Shapiro et al., 2017). Livestock are an important asset and are part of the livelihood for most of the population engaged in mixed crop-livestock farming and pastoral production. The contribution of the livestock sector to the country’s food security and poverty alleviation however remains suboptimal. Disease has been reported to be one of the major constraints to growth of the livestock sector in Ethiopia (FAO, 2015). Animal disease has an important detrimental impact on livestock productivity, resulting in a relatively meagre contribution to food security and the alleviation of poverty.

Addressing the complex challenges facing the livestock sector in Ethiopia requires a critical focus on improvement of veterinary services, specifically the technical, institutional, and regulatory capabilities. With respect to technical capacity, due consideration should be given to enhancing delivery of quality veterinary education with emphasis on improving field and hands-on practical skills of veterinary practitioners. Among the major outcomes of the 2011 evaluation by the OIE for the Performance of Veterinary Services in Ethiopia is the need to strengthen the national animal health workforce and maintain educational and professional quality and standards through continuing professional development (CPD) to improve the quality of veterinary services (FAO, 2018) and maximise both public and private veterinary service delivery.

The Ethiopian Veterinary Association (EVA), in collaboration with stakeholders and partners, has been advocating for strategies that pave the way for veterinary service improvement through policy dialogues, veterinary education, livestock health services, institutional arrangement reforms, and public–private partnership. The EVA recognises that one approach to enhancing the delivery and governance of veterinary services is to deliver skill-based professional training to veterinary practitioners across Ethiopia. Therefore, relevant and priority areas for training must be identified.

The objective of this study was to identify and prioritise areas for training of veterinary professionals in Ethiopia to support veterinary service delivery. Working in collaboration with the African Livestock Productivity and Health Advancement (ALPHA) Initiative, EVA conducted a training needs assessment survey for veterinarians serving the public and private sectors in Ethiopia. The ALPHA Initiative commenced in 2017, co-funded by the partnership of Zoetis Inc. and the Bill & Melinda Gates Foundation (BMGF), with the aim of improving livestock production in Ethiopia, Nigeria, Uganda, and Tanzania (Zoetis 2019). The ALPHA initiative is utilising a bottom-up approach to understand and tackle potential impediments to livestock productivity. The data generated from the survey will identify areas that can be targeted for training and education, allowing for systematic approach to addressing the skills gaps while utilising resources efficiently. Availability of animal health professionals with relevant and applicable skills to local settings will enhance livestock health, welfare, and development in Ethiopia.

## Materials and methods

### Study population

The target study population was veterinarians in Ethiopia. These included veterinarians in government, private practice, and academia. A veterinarian was defined as any individual with a Doctor of Veterinary Medicine (DVM) degree from any of the veterinary faculties in the country or an expatriate veterinarian who practises veterinary medicine in Ethiopia. Approximately 1524 veterinarians were estimated to be involved in the veterinary sector in the country based on a country inventory of the veterinary workforce conducted in 2018 (unpublished data).

### Study approach

A survey of veterinarians in Ethiopia was conducted to collect information on select parameters including demographics; knowledge of antimicrobial resistance (AMR), disease prevention, preventive medicine, epidemiology, disease surveillance, laboratory diagnostic testing, and One Health concept; perceived benefit of training on practical skills, clinical diagnosis and treatment, health management and husbandry practices, and animal welfare; source of information for continuous professional development and preferred format for training delivery; constraints to veterinary service delivery; and opinions about their training needs. In the context of the current study, the One Health concept was defined as acknowledging the close relationships between humans, animals, and ecosystems, and promoting the potential added benefits to each sector or species that emerge as a result of its ‘operationalisation’ (Okello et al., 2014).

The survey was carried out during the EVA conference, held on September 18th–19th, 2019 in Addis Ababa. The questionnaire was presented on tablet computers or iPads using the Qualtrics platform, with the help of six trained assistants. Participants were invited to complete the questionnaire and informed consent was obtained prior to completion of the questionnaire. In addition, a link to the survey was sent electronically to veterinarians registered for the EVA conference via mobile phones and to individuals who were unable to complete the questionnaire on site but who left their contact details. To avoid duplication, an additional question was added to the questionnaire administered online (off the conference site), asking whether the respondent had completed the survey at the conference site. Only individuals that did not previously complete the survey at the conference were allowed to complete the online questionnaire. The survey link was active/available for 10 days after the conference ended.

### Sample size

A convenience sampling method was adopted. A minimum of 300 (30%) veterinarians were anticipated to register and attend the EVA conference and to consent to complete the survey. All veterinarians attending the EVA conference were eligible for inclusion.

### Data analysis

The survey data was downloaded in Microsoft Excel and descriptive statistical analysis was performed. A weighted score was adopted for four questions that asked respondents to rate their knowledge of antimicrobial resistance, epidemiology, disease prevention, and laboratory diagnostic testing. For each question, respondents were asked to score their knowledge from 1 to 5, where 1 represented *very little knowledge* and 5 represented *very knowledgeable* in each of the responses*.* The total score per response was calculated as a *weighted total score* using the formulas reported by Bett et al. (2009) and the weight from 1 to 5 were arbitrarily chosen as done in other studies (Mbuthia et al. 2014; König et al. 2015). The participants’ weighted knowledge score was further reclassified as very low knowledge score (from 0 to 20%), low knowledge score (from 21 to 55%), moderate knowledge score (from 56 to 75%), and high knowledge score (from 76 to 100%).

## Results

A total of 243 respondents completed the survey; 226 veterinarians did so at the conference site and 17 respondents utilised the link to the online survey.

### Respondent demographics

**Fig. 1:**
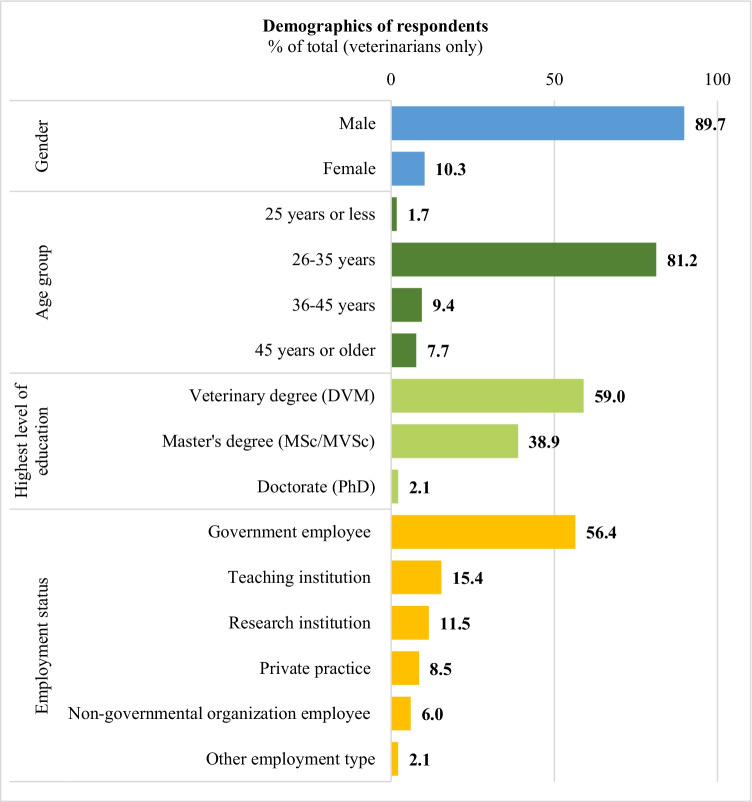
Demographic characteristics of respondents (veterinarians only) (total *n* = 234). Corresponding numbers in each group: males (*n* = 210) and females (*n* = 24); 25 years or less (*n* = 4), 26–35 years (*n* = 190), 36–45 years (*n* = 22) and 45 years or older (*n* = 18); veterinary degree (DVM) (*n* = 138), Master’s degree (MSc/MVSc) (*n* = 91), and Doctorate (PhD) (*n* = 5); government employee (*n* = 132), teaching institution (*n* = 36), research institution (*n* = 27), private practice (*n* = 20), non-governmental organisation employee (*n* = 14), and other employment (*n* = 5). Other employment types reported included drug producer (*n* = 1), supply of veterinary products and training (*n* = 1), higher education institution (*n* = 1), vaccine production (*n* = 1), and veterinary business (*n* = 1)

A total of 96.3% (234/243) of respondents had veterinary degrees; of these, 59% (138/234) had a first degree (DVM), 38.9% (91/234) had an MSc/MVSc, and 2.1% (5/234) had a Ph.D. degree as the highest educational qualification (Fig. 1). Nine respondents (9/243, 3.7%) were not qualified veterinarians (six had a BSc degree, two had an animal health technician certificate, and one had a degree in animal biotechnology); these respondents were not eligible for study inclusion and were therefore removed from the analyses (Supplementary Table 1).

Most of the respondents were male (210/234, 89.7%) and of the age group 26–35 years (190/234, 81.2%). Most of the respondents reported Addis Ababa (91/234, 38.9%) as their usual place of work, while the second and third most frequently reported locations of work were North Shewa (15/234, 6.4%) and Oromia-Finfinnee (10/234, 4.3%), respectively (Supplementary Table 2). Most respondents 56.4% (132/234) were government employees; 15.4% (36/234) worked in teaching, 11.5% (27/234) worked in research institutions, 8.5% (20/234) in private practice, 6% (14/234) in non-governmental organisations, and 2.1% (5/234) had other employment (e.g., pharmaceutical products production or distribution) (Fig. 1).

**Fig. 2:**
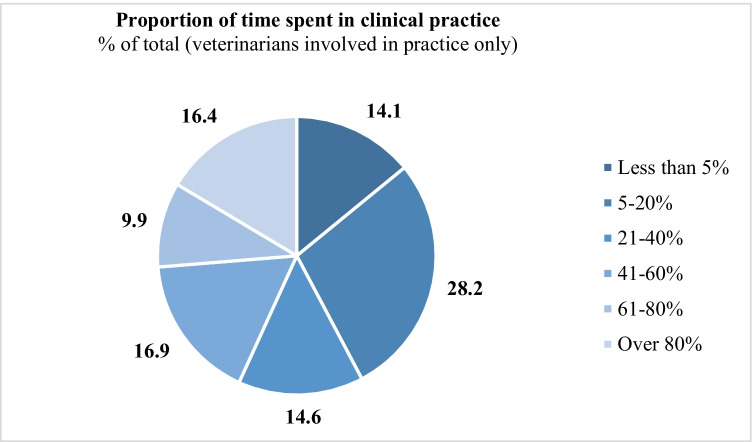
Proportion of working hours reportedly spent in clinical practice by veterinarians in Ethiopia (only those who were involved in clinical practice; total *n* = 213). Corresponding numbers in each category: less than 5% (*n* = 30), 5–20% (*n* = 60), 21–40% (*n* = 31), 41–60% (*n* = 36), 61–80% (*n* = 21), over 80% (*n* = 35)

Overall, 91% (213/234) of respondents were involved in clinical practice. Within this group, the highest number of respondents (60/213, 28.2%) spent between 5 and 20% of their working time in clinical practice, and 16.4% (35/213) spent over 80% of their working time in clinical practice (Fig. 2).

Those respondents involved in clinical practice (*n* = 213) were asked to rank five clinical areas, based on the time spent working on activities in each of these areas. Of those who responded to these questions (*n* = 174), the majority spent most time performing preventive medicine activities (such as vaccination, deworming, and others) (81/174, 46.6%), followed by medical treatment (72/174, 41.4%). By contrast, a large proportion of respondents spent the least amount of time performing surgical interventions (78/174, 44.8%), followed by activities related to routine husbandry (47/174, 27%) and reproduction (40/174, 23%). The full results on the ranking of each of the five clinical areas are displayed in Supplementary Table 3.

### Knowledge 

#### Antimicrobial resistance

**Fig. 3:**
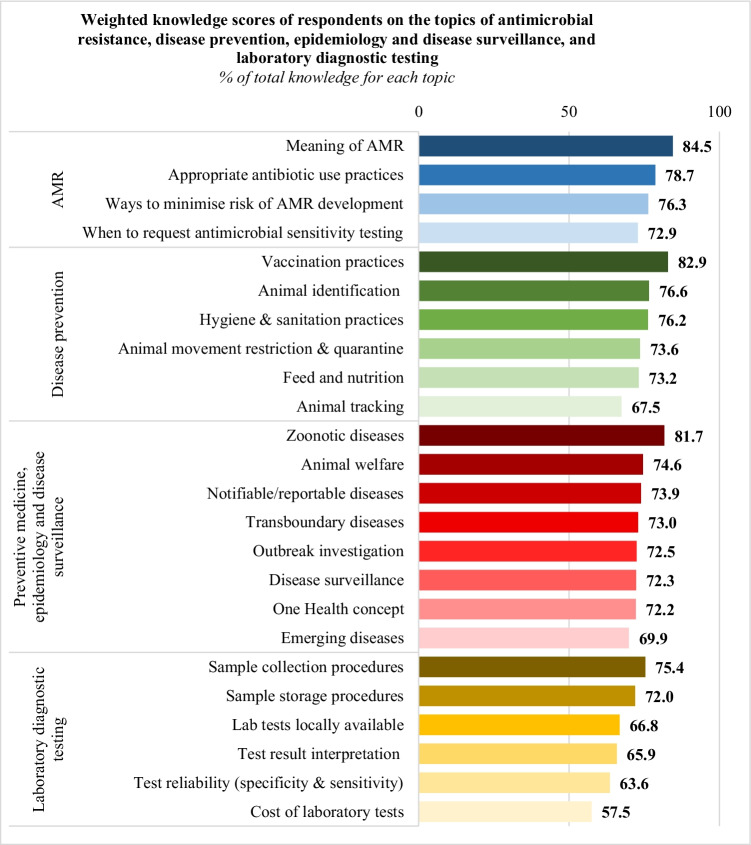
Weighted knowledge scores of respondents for each aspect of the listed topics (total *n* = 234, except for ‘Animal tracking’ *n* = 233)

The total weighted AMR knowledge score was 78.1%; the score ranged between moderate and high depending on the specific aspects of AMR, with the lowest score reported for *when to request AST* (853/1170, 72.9%) (Fig. 3).

#### Disease prevention

The total weighted knowledge score on disease prevention was 75% and it ranged between moderate and high (67.5 to 82.9%), with the lowest weighted scores reported for *animal tracking* (786/1165, 67.5%), *feed and nutrition* (856/1170, 73.2%), and *animal movement restriction and quarantine* (861/1170, 73.6%) (Fig. 3).

#### Preventive medicine, epidemiology, and disease surveillance

The total weighted knowledge score was 73.8%; based on the different aspects of this topic, the scores were moderate to high, with the lowest score reported for *emerging diseases* (818/1170, 69.9%), *One Health concept* (845/1170, 72.2%), *disease surveillance* (846/1170, 72.3%), and *outbreak investigation* (848/1170, 72.5%) (Fig. 3).

#### Laboratory diagnostic testing

The total weighted knowledge score was moderate (66.9%), with lowest scores for *cost of laboratory tests* (673/1170, 57.5%), *test reliability (specificity and sensitivity)* (744/1170, 63.6%), and *test result interpretation* (e.g., interpreting poultry antibody titres) (771/1170, 65.9%) (Fig. 3).

Most respondents (72.6%, 170/234) reported being aware of a national disease reporting system in Ethiopia and 27.4% (64/234) were not aware.

### Perceived benefit of training on practical skills, clinical diagnosis and treatment, health management and husbandry practices, and animal welfare

Most respondents would like to receive training on disease of the following animal species: cattle (155/234, 66.2%), poultry (134/234, 57.3%), and sheep and goats (97/234, 41.5%) (Supplementary Table 4).

The perceived benefits of receiving training on practical skills, clinical diagnosis and treatment, herd health management and husbandry practices, and animal welfare were assessed and scored.

**Fig. 4:**
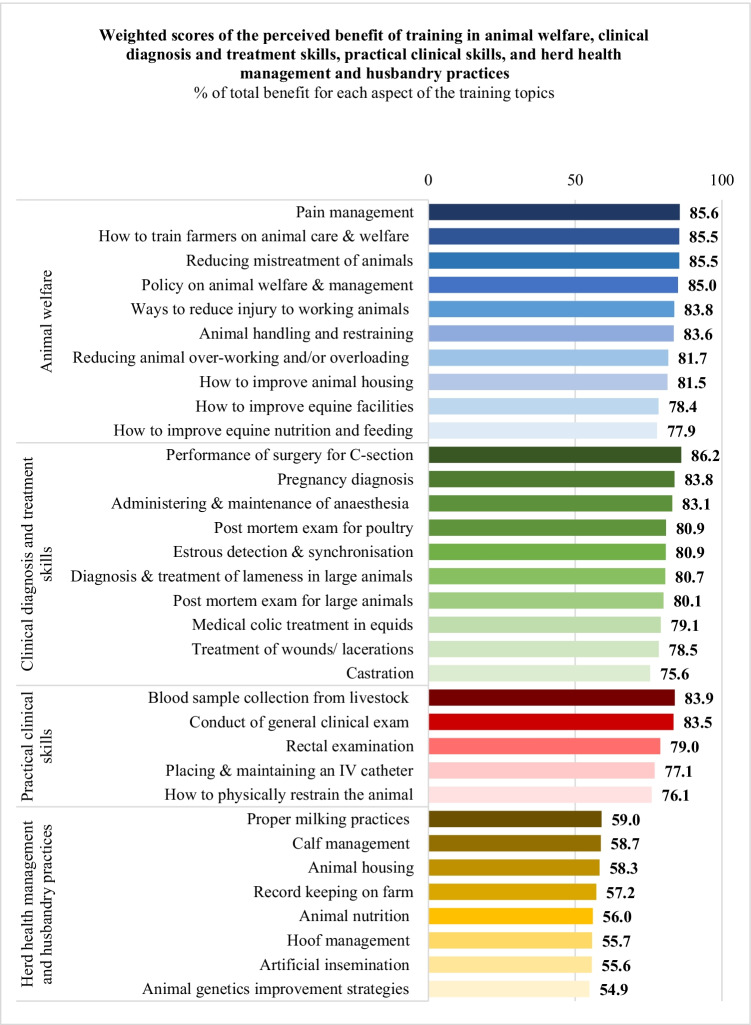
Weighted training benefit scores of respondents for each aspect of the listed training topics (total *n* = 234, except for ‘How to physically restrain the animal’ *n* = 233, ‘Placing & maintaining an IV catheter’ *n* = 233, and ‘Rectal examination’ *n* = 233)

For animal welfare, the training areas considered most beneficial were *pain management* (85.6%, 1002/1170), *how to train communities and farmers on animal care and welfare* (85.5%, 1000/1170), *reducing mistreatment of animals* (85.5%, 1000/1170), and *legal frameworks/policies on animal welfare and management* (85%, 995/1170) (Fig. 4).

For clinical diagnosis and treatment, the training areas considered most beneficial were *how to perform a caesarean Sect. *(86.2%, 1008/1170), *pregnancy diagnosis* (83.8%, 981/1170), and *administration and maintenance of local anaesthesia* (83.1%, 972/1170) (Fig. 4).

For practical clinical skills, the training areas considered most beneficial were *blood sample collection* (83.9%, 982/1170), *conduct of general clinical exam* (83.5%, 977/1170), and *rectal examination* (79%, 920/1165) (Fig. 4).

For herd health management and husbandry practices, there was a general drop in the perceived benefit. The percent of total weighted benefit ranged between only 54.9% for *animal genetics improvement strategies* (642/1170) and 59% for *proper milking practices* (690/1170) (Fig. 4).

The top topics that respondents regarded as most beneficial to receive refresher training on were training in *laboratory diagnostics* (165/234, 70.5%), followed by *antimicrobial resistance and antibiotic sensitivity testing* (153/234, 65.4%), and *diagnosis and treatment of common livestock diseases* (143/234, 61.1%) (Supplementary Table 5).

### Source of information for continuous development and preferred format for training delivery

Most of the respondents (171/234, 73.1%) preferred using reports or publications to keep up to date with current information on veterinary affairs. Other sources of information included conferences (135/234, 57.7%), refresher trainings (134/234, 57.3%), post-graduate training (102/234, 43.6%), continuous professional development (94/234, 40.2%), and word of mouth (46/234, 19.7%). Other ways of keeping updated with current information were reported by 6/234 (2.6%) participants; these included using the internet/online (including social media and online courses) (4/234, 1.7%), short-term trainings (1/234, 0.4%), and veterinary related news (1/234, 0.4%).

The most preferred format for the delivery of training was practical workshops (180/234, 76.9%), followed by face-to-face theory lectures (127/234, 54.3%), external training or training that takes place outside a participant’s usual place of work (121/234, 51.7%), in-practice training (114/234, 48.7%), online courses (64/234, 27.4%), social media platforms (34/234, 14.5%), conferences (29/234, 12.4%), and reports/guidelines (18/234, 7.7%).

### Constraints to veterinary service delivery

The most frequently reported constraints to the delivery of veterinary services were lack of appropriate tools and equipment (187/234, 79.9%), unavailability of sufficient funds from the government (182/234, 77.8%), lack of access to diagnostic laboratories (180/234, 76.9%), and unavailability of diagnostic laboratories (158/234, 67.5%). Other reported challenges were the animal health and welfare policies that were in place (120/234, 51.3%), lack of skilled staff (114/234, 48.7%), and few personnel working at the practice (60/234, 25.6%). Other constraints (3/234, 1.3%) included laboratory kit costs, lack of focus by the government, and lack of political commitment (each of these was reported by one participant).

## Discussion

Most respondents were male, aged 26–35 years. This may be a reflection of veterinary professionals that attended the EVA conference, but not necessarily a reflection of the entire population of veterinary professionals in Ethiopia. The reasons for the gender imbalance in the respondent veterinarians are not clear, but this finding suggests more should be done to encourage females to become animal health professionals and having a high proportion of respondents fall in the age bracket of 26–35 years may indicate this is a growing profession or that fewer older veterinarians attend the EVA conference.

Most respondents were located in Addis Ababa. This is not unexpected since the survey was administered at the EVA conference held in Addis Ababa and proximity to the conference site might have played a role in attendance. In addition, commercial livestock farms are mainly concentrated in and around Addis Ababa and this might explain a high number of respondents operating in the area surrounding the capital (Admassu, 2003). In terms of employment, most veterinarians were employed by the government (56.4%) and only a minority in private practice (8.5%). This is in line with the findings of Jibat et al. (2015) that reported over half of veterinary services in central Ethiopia are delivered by the public sector.

Most participants reported being involved in clinical practice (91%), but the amount of time spent in clinical activities varied widely and just 26.3% of respondents were involved in clinical practice for over 60% of their time. Most veterinarians involved in clinical practice focussed on preventive medicine (vaccination, deworming, etc.) and medical treatment, but less on reproduction, routine husbandry, and surgery. Hadush (2015) reported that veterinarians, especially the government employees, were involved in several activities such as provision of vaccination and treatment services, meat inspection, and reporting of animal disease occurrence, while veterinarians in the private sector were involved in clinical practice but also operated drug shops and were involved in the importation of veterinary products (Hadush, 2015).

The self-rated knowledge of participants on antimicrobial resistance, epidemiology, disease prevention, and laboratory diagnostic testing, ranged from moderate (from 55 to 75% of total score) to high (from 76 to 100% of total score). The lowest knowledge areas were laboratory testing, mainly knowledge of the laboratory tests locally available, interpretation of test results, understanding of test sensitivity and specificity, and costs of laboratory tests. The low knowledge on available laboratory tests may be linked to a reduced or lack of access to diagnostic laboratories. Almost 77% of the respondents reported that accessing a diagnostic laboratory is challenging and over 67% reported that laboratories are unavailable in their work areas. In 2016, only five regional laboratories were reported in the country and two of the five were almost inactive, and clinicians reported rare use of the laboratory (Hooper, 2016).

A moderate weighted knowledge was reported for disease prevention, epidemiology, and disease surveillance (from 55 to 75% of total score). For disease prevention, the lowest knowledge was reported for animal movement, quarantine, tracking, and nutrition. A better understanding of animal and movement tracing system by animal health workers may help reduce livestock economic losses (Elbakidze, 2015). Having a well-structured system to track livestock movements helps in investigating risk factors for disease transmission and in planning interventional strategies (Mekonnen et al., 2019). Therefore, establishment of a functioning animal movement and tracking system that is well understood by veterinary professionals may help improve disease prevention.

Almost one-third of the respondents (27.4%) did not know that there is a National Disease Reporting System in Ethiopia, and their overall knowledge on issues such as disease surveillance, emerging, transboundary, and notifiable or reportable diseases was moderate. A National Animal Disease Surveillance System (NASDS) is used in Ethiopia and alternatives to paper recording to ease communications have been explored, such as smartphone-based applications (Beyene et al., 2018). Nonetheless, the data from NASDS, the Animal Disease Notification and Identification System (ADNIS), and Livestock Identification and Traceability System (LITS) may not be up to date and not readily available for analysis (European Union, 2017). Engaging operators and specialists in the animal health sector in training concerning disease reporting and surveillance would raise the awareness of the importance and impact of timely and effective communication of findings in order to contain spread of disease outbreaks.

Respondents’ knowledge on AMR was moderate to high (72.9 to 84.5%) with the lowest score (72.9%) reported for how to request antimicrobial sensitivity testing (AST). The use and correct interpretation of AST is essential to the selection of antibiotics that can be effective against a specific pathogen. The appropriate selection and use of antibiotics could help reduce the risk of development of antimicrobial resistance.

The main three topics the participants were interested receiving training on were laboratory diagnostics (70.5% of participants), AMR and AST use (65.4% of participants), and diagnosis and treatment of common livestock diseases (61.1% of participants). Targeted training on laboratory diagnostics, AMR, and AST use may help address the perceived gaps in knowledge highlighted by the respondents, thus improving the veterinarians’ knowledge on the optimal test to run for disease diagnosis, laboratory test result interpretation, and appropriate antibiotic to select for treatment.

Other topics considered beneficial were basic epidemiology (56.4%) and practical clinical skills, such as blood sample collection and general clinical examination (49.6%). The conduct of general clinical examination and performance of simple clinical procedures (blood sampling, rectal examination, placing intravenous catheter, etc.) are standard practice in the clinical sector. The clinical procedures most frequently selected by respondents were performance of caesarean section, diagnosis of pregnancy, oestrous detection, and anaesthetic procedures. Refresher training in these areas will equip veterinarians with relevant skills to enable evidence-based clinical decisions.

Training on animal welfare was selected less frequently as only 20.9% selected this response. On the contrary, weighted knowledge scores for animal welfare were high (between 72.3 and 82.1%). These findings suggest that respondents may understand the impact poor animal welfare practices can have on livestock performance and prefer having training on other topics.

The three main animal species respondents would like to receive training in relation to animal diseases were cattle (66.2%), poultry (57.3%), and sheep and goats (41.5%). The species preferences reflect the most common type of livestock present in the country. In 2015, 56,706,390 cattle, 56,866,719 chickens, and 58,445,335 small ruminants were estimated in Ethiopia (FAO, 2017).

While participants mainly used reports or publications (73.1%) to keep up to date with current information on veterinary medicine, the preferred format for training was practical workshops (76.9%). Other popular formats were face-to-face theory lectures (54.3%) and external training or training held outside a participant’s usual place of work (51.7%). These findings outline how human and practical component of training are cherished by potential trainees. Previous studies have shown that trainees undertaking face-to-face training have shown a higher level of satisfaction compared with individuals on distant training (Bernard et al., 2004). However, it has also been shown that in terms of knowledge gain, distant learning and face-to-face learning do not differ much, either for short-term or long-term knowledge (Olivet et al., 2016). Additionally, when applicable, the practical component plays a crucial part in training, enhancing the content of theoretical lecturing with real applications and strengthening the learning process (Katajavuori et al., 2006).

The key constraints reported in the current study to negatively impact veterinary services were lack of appropriate tools and equipment and lack or unavailability of access to diagnostic laboratories and funds. These findings agree with previous studies that explored the challenges of the Ethiopian veterinary sector. A shortage of laboratory facilities and materials and inadequate funding were reported as weaknesses of the government and private veterinary sectors in Ethiopia (Kebede et al., 2014; Jibat et al., 2015). Providing access to adequately facilitated laboratories is crucial to the improvement of veterinary services and privatisation and improvements in the current private sector are necessary to encourage sustainability.

A few study limitations were observed in the current study. Although the findings help in underlining the participants’ areas of low knowledge, potential topics for training, and constraints to the veterinary sector, it is important to note that results were generated from a sample of participants that were not randomly selected. Therefore, caution should be exercised in generalising the findings to the entire population of Ethiopian animal health professionals. Secondly, because the survey was undertaken at a conference, and participants were not randomly selected from the list of all registered and eligible veterinarians in the country, it is likely that individuals with interest in continuous professional development were overrepresented. Additionally, veterinarians with higher economic resources or more closely located to the conference may have been more likely to participate compared to veterinarians with low resources or based far away from Addis Ababa, and this might have biased the sample. Thirdly, as knowledge was self-assessed by participants, it is likely biased towards higher knowledge levels. Lastly, it is possible that the structure of questions requiring ranking or use of the Likert scale may have been unfamiliar to respondents and may have been difficult to answer. However, this risk was mitigated through pre-testing of the questionnaire, further refining of questions and access to trained facilitators at the conference.

In conclusion, the study findings suggest the following areas are relevant and can be prioritised when considering CPD to strengthen the skills of animal health professionals in Ethiopia: laboratory diagnostics and testing, including test cost and knowledge of local availability of laboratory tests, costs, result interpretation (test specificity and sensitivity), and sample storage procedures; knowledge on appropriate use of antimicrobial sensitivity testing and how to minimise AMR; diagnosis and treatment of main diseases of cattle, poultry, sheep, and goats; basic epidemiology including disease surveillance, outbreak investigation, transboundary, emerging, zoonotic, notifiable/reportable diseases, animal tracking, and animal movement restriction and quarantine; basic clinical skills including conduct of general examination, blood sampling, and rectal examination; and advanced clinical procedures including caesarean section or diagnosis and treatment of lameness in ruminants, camels, and equids.

In addition, there are other relevant information to consider in relation to improving the skills of animal health professionals in Ethiopia. The preferred format for delivery of training was practical workshops, face-to-face lectures, and external training or training held outside a participant’s usual place of work. It is important to consider and vary the format of training based on feasibility and geographic location of target participants across the country. Secondly, the constraints within the veterinary sector including lack of facilities, equipment, and resources must be considered in order to provide both the knowledge and the means for a better delivery of veterinary services. Providing adequate laboratory facilities and materials should also be included in future interventional strategies. Thirdly, as no studies were previously conducted to address the key training needs of veterinary practitioners in Ethiopia, this survey could provide baseline information on priority training areas for veterinary professionals working at different levels across the country. Most importantly, the findings could potentially contribute to national efforts to develop and implement CPD programme in the veterinary domain, in view of ensuring better and rationalised veterinary service delivery. Lastly, it is recommended that concerned key stakeholders in veterinary service delivery led by EVA should play their roles to deliver quality training on the prioritised topics.

## Supplementary Information

Below is the link to the electronic supplementary material.

Supplementary file1 (PDF 138 KB)
