# Supplementary material for: Training needs assessment of veterinary practitioners in Ethiopia
Source: Trop Anim Health Prod. 2022 Jan 22;54(1):72. doi: 10.1007/s11250-022-03075-0 (PMC8783848; doi:10.1007/s11250-022-03075-0)
Supplement: Supplementary file 1 — Supplementary file1 (PDF 138 KB) [file 11250_2022_3075_MOESM1_ESM.pdf]

Supplementary Tables

Supplementary Table 1. Education levels of respondents.

Variable	Response	n	Percent
Highest level of education you attained – all respondents (**n=243**)	Veterinary degree (DVM)	138	56.8
	Master's degree (MSc/MVSc)	91	37.4
	Bachelor's degree (B.Sc.)	6	2.5
	Doctorate (PhD)	5	2.1
	Animal Health Technician (certificate)	2	0.8
	Other, please specify	1	0.4
Highest level of education you attained – only veterinarians (**n=234**)	Veterinary degree (DVM)	138	59.0
	Master's degree (MSc/MVSc)	91	38.9
	Doctorate (PhD)	5	2.1

Supplementary Table 2. Zone of usual place of work for respondents (n=234).

Response	N=234	%
Addis Ababa	91	38.9
North Shewa	15	6.4
Other	11	4.7
Oromia-Finfinne	10	4.3
West Shewa	9	3.8
East Welega	8	3.4
West Welega	8	3.4
Wolayita	8	3.4
Arsi	7	3.0
East Shewa	7	3.0
Gamo Gofa	6	2.6
Jimma	6	2.6
Debub bete-Amhara (Wollo) Zone / South Bete-Amhara zone	5	2.1
Southwest Shewa	5	2.1
East Hararghe	4	1.7
Harari	4	1.7
Mekele	4	1.7
Fafan	2	0.9
Gurage	2	0.9
Illubabor Zone	2	0.9
Jimma	2	0.9
Kelam Welega	2	0.9
Kembata Tembaro	2	0.9
Adama	1	0.4
Administrative Zone 1 (i.e. Awsi Rasu)	1	0.4
Administrative Zone 4 (i.e. Fantena Rasu)	1	0.4
Anuak	1	0.4
Bahir Dar (special zone)	1	0.4
Bale	1	0.4
Bench Maji	1	0.4
Borana	1	0.4
Dawro	1	0.4
Dire Dawa	1	0.4
Hadiya	1	0.4
Jarar	1	0.4
North Gondar	1	0.4
West Haraghe	1	0.4

Supplementary Table 3. Ranking of five clinical areas based on the time spent in clinical practice (n=213) working on listed activities.

	**Response (n=174)**										
	**1st position**		**2nd position**		**3rd position**		**4th position**		**5th position**		**Total**
	**n**	**%**	**n**	**%**	**n**	**%**	**n**	**%**	**n**	**%**	**n**
Preventive medicine (vaccination, worming, nutritional advice etc.)	81	46.6	53	30.5	23	13.2	14	8.0	3	1.7	174
Reproduction (pregnancy diagnosis, breeding etc.)	8	4.6	25	14.4	45	25.9	56	32.2	40	23.0	174
Routine husbandry (foot trimming, castration of young stock etc.)	5	2.9	23	13.2	58	33.3	41	23.6	47	27.0	174
Medical treatment (antibiotics etc.)	72	41.4	59	33.9	19	10.9	18	10.3	6	3.4	174
Surgical intervention (rumenotomy, caesarean section etc.)	8	4.6	14	8.0	29	16.7	45	25.9	78	44.8	174

Supplementary Table 4. Animal species on which the respondents would like to receive training on regarding animal diseases (n=234)

**Response**	**n**	**%**
Cattle	155	66.2
Poultry	134	57.3
Sheep and goats	97	41.5
Horses and donkeys	74	31.6
Small animals (e.g. dogs, cats)	71	30.3
Fish	41	17.5
Camels	28	12.0
Pigs	15	6.4
Other, please specify	4	1.7

Other responses (n=4) included: all (n=1), companion animals (n=1), honey bee (n=1), and veterinary drug handling (n=1). Respondents selected all responses that apply, so percentages do not add up to 100%.

Supplementary Table 5. Topics indicated by the respondents as most beneficial to receive refresher trainings on (n=234).

Response	n	%
Laboratory diagnostics	165	70.5
AMR & antibiotic sensitivity testing	153	65.4
Diagnosis & treatment of common livestock diseases	143	61.1
Basic epidemiology	132	56.4
Disease surveillance & reporting	117	50.0
Practical clinical skills	116	49.6
Biosecurity practices	114	48.7
Vaccination practices	95	40.6
New veterinary products & tools for disease treatment	90	38.5
Animal welfare	49	20.9
Other topic	2	0.9

Other topics (n=2) included: legal framework on animal disease (n=1) and veterinary drug regulation related trainings (n=1). Respondents selected multiple responses (their top five choices), so percentages do not add up to 100%.
